# Hemodialysis Impact on Motor Function beyond Aging and Diabetes—Objectively Assessing Gait and Balance by Wearable Technology

**DOI:** 10.3390/s18113939

**Published:** 2018-11-14

**Authors:** He Zhou, Fadwa Al-Ali, Hadi Rahemi, Nishat Kulkarni, Abdullah Hamad, Rania Ibrahim, Talal K. Talal, Bijan Najafi

**Affiliations:** 1 Interdisciplinary Consortium on Advanced Motion Performance (iCAMP), Michael E. DeBakey Department of Surgery, Baylor College of Medicine, Houston, TX 77030, USA; he.zhou2@bcm.edu (H.Z.); hrahemi@gmail.com (H.R.); nishat.kulkarni@bcm.edu (N.K.); 2 Fahad Bin Jassim Kidney Center, Department of Nephrology, Hamad General Hospital, PO Box 3050 Doha, Qatar; falali1@hamad.qa (F.A.-A.); ahamad9@hamad.qa (A.H.); ribrahim4@hamad.qa (R.I.); 3 Diabetic Foot and Wound Clinic, Hamad Medical Co, PO Box 3050 Doha, Qatar; ttalal@hamad.qa

**Keywords:** hemodialysis, end stage renal disease, diabetes, motor performance, gait, balance, wearables, aging, frailty, diabetic peripheral neuropathy, falls

## Abstract

Motor functions are deteriorated by aging. Some conditions may magnify this deterioration. This study examined whether hemodialysis (HD) process would negatively impact gait and balance beyond diabetes condition among mid-age adults (48–64 years) and older adults (65+ years). One hundred and ninety-six subjects (age = 66.2 ± 9.1 years, body-mass-index = 30.1 ± 6.4 kg/m^2^, female = 56%) in 5 groups were recruited: mid-age adults with diabetes undergoing HD (Mid-age HD+, n = 38) and without HD (Mid-age HD−, n = 40); older adults with diabetes undergoing HD (Older HD+, n = 36) and without HD (Older HD−, n = 37); and non-diabetic older adults (Older DM−, n = 45). Gait parameters (stride velocity, stride length, gait cycle time, and double support) and balance parameters (ankle, hip, and center of mass sways) were quantified using validated wearable platforms. Groups with diabetes had overall poorer gait and balance compared to the non-diabetic group (*p* < 0.050). Among people with diabetes, HD+ had significantly worsened gait and balance when comparing to HD− (Cohen’s effect size *d* = 0.63–2.32, *p* < 0.050). Between-group difference was more pronounced among older adults with the largest effect size observed for stride length (*d* = 2.32, *p* < 0.001). Results suggested that deterioration in normalized gait speed among HD+ was negatively correlated with age (*r* = −0.404, *p* < 0.001), while this correlation was diminished among HD−. Interestingly, results also suggested that poor gait among Older HD− is related to poor ankle stability, while no correlation was observed between poor ankle stability and poor gait among Older HD+. Using objective assessments, results confirmed that the presence of diabetes can deteriorate gait and balance, and this deterioration can be magnified by HD process. Among HD− people with diabetes, poor ankle stability described poor gait. However, among people with diabetes undergoing HD, age was a dominate factor describing poor gait irrespective of static balance. Results also suggested feasibility of using wearable platforms to quantify motor performance during routine dialysis clinic visit. These objective assessments may assist in identifying early deterioration in motor function, which in turn may promote timely intervention.

## 1. Introduction

Motor function, such as gait and balance ability, is the major determinant of an independent and productive life [1]. Gait and balance are essential for predicting poor quality of life, morbidity, and mortality [2,3]. Aging causes deterioration in the sensory systems and changes the pattern of muscle activity, leading to degradation in gait and balance [4,5,6]. In addition, chronic disease, such as diabetes mellitus (DM) and end stage renal disease (ESRD), could accelerate this degradation. For people with diabetes and ESRD undergoing hemodialysis (HD) process, degradation in gait and balance may be even worse [7,8,9]. These patients are often required to visit dialysis clinic 3 time per week and spend 4 h each time to receive HD process. After HD process, they are often exhausted, limiting their ability to be physical active. Long immobility may lead to muscle loss, which in turn may deteriorate motor function. Without timely intervention, motor function deterioration among HD patients may lead to serious adverse outcomes, including foot ulcer, amputation, early frailty, risk of falling, and loss of independency, which may further complicate their conditions. Together, with the increasing HD population [10], it imposes huge burden to the health care system [11].

A few previous studies compared motor performance of HD patients with healthy controls [7,8,9], but these studies suffer from several shortcomings limiting the understanding of negative effect of HD on motor performance beyond diabetes and aging. Some of the limitations include self-reported bias, semi-objective inaccuracies, focusing on only gait or only static balance, as well as lack of comparison between people with diabetes undergoing HD and without HD. Due to the prolonged HD process, post-dialysis exhaustion, limitation of transportation to research facilities, as well as immobility caused by HD, it is often impractical to bring HD patients, in particular older HD patients, to a dedicated gait laboratory for study [12]. Even a study could be conducted in the gait laboratory, the results may still be biased since the study sample is limited to non-cohort selected HD population (those with better mobility and cognitive function who can visit a gait laboratory).

Recent advances in wearable technologies have opened new opportunities to objectively assess motor performance in place anytime and anywhere [13,14,15,16,17,18]. Smart watches, smart pendants, and other smart wearables or mobile based applications already marketed to the young and healthy population will take an over-growing presence in the patient care marketplace, including screening motor degradation in response to different conditions such as frailty, diabetes, Parkinson’s, etc. [19,20]. Wearable devices can track nearly everything, from early stroke detection, to monitoring physiological parameters, quantifying physical activity, monitoring sleep quality, determining gait structures, and measuring plantar pressures and shear [21,22,23,24,25]. The key advantage of wearable sensors is that no dedicated lab environment or infrastructure is required to extract parameters of interest. As a result, motor function assessments, such as gait and balance tests, can be performed in any clinical setting and during routine clinic visit. This is a key advantage for screening motor performance in people undergoing hemodialysis as often they have limited mobility and suffer from post-dialysis fatigue and thus unmotivated to visit any place different than their routine dialysis clinic. Because of these limitations, little is known about the impact of HD on degradation of motor function. We believe “we can’t manage, what we can’t measure”. Thus wearable sensors may offer a practical solution to routinely screen degradation in motor performance caused by HD process and potentially assist with timely intervention to limit its consequences. In this study, we proposed using wearable sensors to objectively quantify gait and balance performances during a regular dialysis clinic visit. We proposed to determine the magnitude of gait and balance deteriorations potentially caused by HD process beyond aging and diabetes condition. To examine the degree of degradation in motor performance caused by HD, we compared gait and balance performances among people with diabetes undergoing HD to those with diabetes but no HD, as well as to age-matched non-diabetic controls. The hypotheses of this study were: (1) compared to age-matched non-diabetic controls, people with diabetes have poorer gait and balance irrespective of HD process; (2) HD magnifies decline in gait and lower body joints stability irrespective of age; and (3) along with aging, the degradation in gait and lower body joints stability among HD patients are magnified.

## 2. Methods

### 2.1. Study Population

One hundred and ninety-six eligible subjects were recruited in this study: 78 mid-age (48–64 years old) adults with diabetes (‘Mid-age DM+’), 73 older (65–90 years old) adults with diabetes (‘Older DM+’), and 45 older (65–88 years old) non-diabetic controls (‘Older DM−’). Furthermore, based on ESRD/HD condition, Mid-age DM+ group was further classified into ‘Mid-age HD−’ (n = 40) and ‘Mid-age HD+’ (n = 38) groups. Similarly, Older DM+ groups was further classified into ‘Older HD−’ (n = 37) and ‘Older HD+’ (n = 36) groups. Subjects were excluded from the study if they were non-ambulatory, had severe gait or balance problem (e.g., unable to walk a distance of 15 m independently with or without assistive device or unable to stand still without moving feet), or were unwilling to participate. All subjects signed a consent form for this study. This study was approved by the local institutional review boards (IRB). All participates in the study read and signed the IRB approved forms of informed consent before initiation of any assessment or data collection.

### 2.2. Demographic and Clinical Information

Subjects’ demographics including age, gender, body-mass-index (BMI), and fall history were collected. All subjects underwent clinical assessments, including Fall Efficacy Scale—International (FES-I) [26], Center for Epidemiologic Studies Depression scale (CES-D) [27], and Physical Frailty Phenotype [28]. Subject with diabetes also underwent Vibration Perception Threshold test (VPT) [29], Ankle Brachial Index test (ABI) [30], and glycated hemoglobin test (HbA1c) [31]. The FES-I and its cutoff score, as suggested by Delbaere et al. [32], were used to identify subjects with high concern about falling. The CES-D short-version scale was used to measure self-reported depression symptoms. A cutoff of CES-D score of 16 or greater was used to identify subjects with depression [33]. The Physical Frailty Phenotype, including unintentional weight loss, weakness (grip strength), slow gait speed (15-foot gait test), self-reported exhaustion, and self-reported low physical activity, was used to assess frailty [28]. Subjects with 1 or 2 positive criteria were considered pre-frail, and those with 3 or more positive criteria were considered frail. Subjects negative for all criteria were considered robust [28]. Plantar numbness was evaluated by the VPT measured on six plantar regions of interest, including the left and right great toes, 5th metatarsals, and heels. A subject was designated with Diabetic Peripheral Neuropathy (DPN) if his/her measured VPT value for any of the six plantar regions of interests reached 25 volts or greater [34]. The ABI was calculated as the ratio of the systolic blood pressure measured at the ankle to the systolic blood pressure measured in the upper arm. A subject was designated with Peripheral Artery Disease (PAD) if his/her ABI value was either greater than 1.2 or smaller than 0.8 [35].

### 2.3. Gait Test

**Figure 1:**
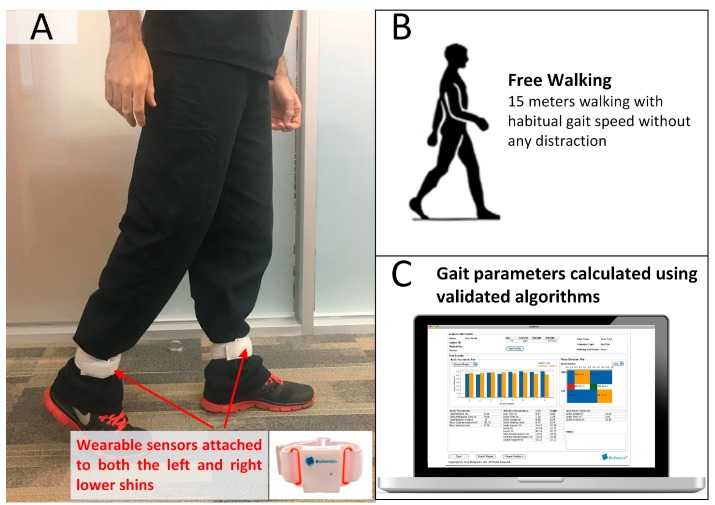
An Illustration of gait test. (**A**) Two wearable inertial sensors were attached to left and right lower shins. (**B**) The subject was asked to walk with habitual gait speed for 15 m. (**C**) Using validated algorithms gait parameters of interest, including stride velocity (unit: m/s), normalized stride velocity to height (unit: height/s), stride length (unit: m), normalized stride length to height (unit: % height), gait cycle time (unit: s), and double support (unit: %) were calculated.

For all subjects, two wearable sensors (LegSys™, BioSensics, Watertown, MA, USA) were attached to left and right lower shins to quantify gait parameters of interest (Figure 1). Each LegSys^TM^ sensor equips with a triaxial accelerometer (±2 g) and triaxial gyroscope (±2000 deg/s) to estimate spatio-temporal parameters of gait. The signals were digitized at a sampling frequency of 100 Hz and transmitted to the LegSys™ software installed on a standard computer or tablet by real-time Bluetooth transmission. The method for calculation of the spatio-temporal parameters of gait was described in detail elsewhere [36,37]. Subjects were asked to walk with their habitual gait speed for 15 m as suggested in previous studies [38,39] without any distraction. Gait parameters, including stride velocity (unit: m/s), normalized stride velocity to height (unit: height/s), stride length (unit: m), normalized stride length to height (unit: % height), gait cycle time (unit: s), and double support (unit: %), were calculated during steady state phase of walking using validated algorithms [38,40]. The initiation of gait steady state was objectively estimated using a validated algorithm described elsewhere [41].

### 2.4. Balance Test

**Figure 2:**
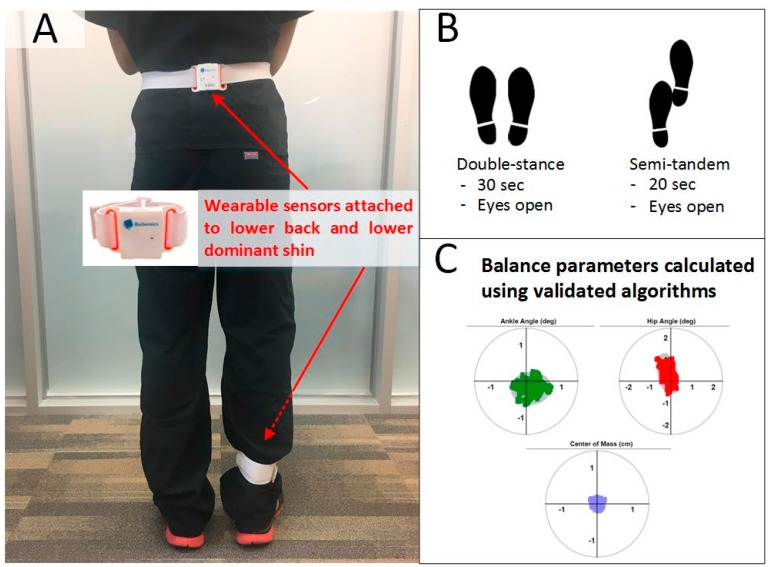
An Illustration of balance test. (**A**) Two wearable inertial sensors were attached to lower back and lower dominant shin. (**B**) Double-stance for 30 s and semi-tandem for 20 s under eyes open condition were performed. (**C**) Using validated algorithms balance parameters of interest, including ankle sway (unit: deg^2^), hip sway (unit: deg^2^), center of mass sway (unit: cm^2^), and normalized center of mass sway to height (unit: cm^2^/height) were calculated.

The same wearable sensors used in the gait test were attached to the lower back and lower dominant shin to measure balance performances by a two-link model (Figure 2). The two sensors can estimate three-dimensional angles of the hip and ankle joints. Each sensor provide real-time (sample frequency 100 Hz) quaternions (qw, qx, qy, qz) that are subsequently converted to Euler angles denoted as θ, φ, and ψ. The resulting three-dimensional angles are used to estimate the trajectory of the subject’s ankle and hip [42]. A two-segment model (upper body rotation around hip and lower body rotation around ankle) was used to calculate center of mass (CoM) motion in anterior–posterior (AP) and medial–lateral (ML) directions using the estimated ankle and hip angles and subject’s anthropometric data (weight and height) [42]. Double-stance quiet standing balance test for 30 s under eyes open condition was performed for all subjects. In addition, semi-tandem balance test was also performed for 20 s under eyes open condition for the groups with diabetes. Since some of the subjects were unable to perform balance test under eyes closed condition, we limited this study to eyes open condition. In the double-stance test, the subject stood in the upright position, keeping feet close together but not touching, with arms folded across the chest. In the semi-tandem test, the subject stood with the dominant foot a half-foot behind the other, keeping feet close together but not touching, with arms folded across the chest. Balance parameters, including ankle sway (unit: deg^2^), hip sway (unit: deg^2^), center of mass sway (unit: cm^2^), and normalized center of mass sway to height (unit: cm^2^/height) were calculated using validated algorithms [43].

### 2.5. Statistical Analysis

All continuous data were presented as mean ± standard deviation. All categorical data were expressed as count (percentage). The Shapiro-Wilk test was applied to test normality of data. Analysis of covariance (ANOVA) was used to compare between-group gait and balance performances, with adjustment for age, gender, BMI, and maximum VPT value. Fisher’s least significant difference-based post-hoc test was performed for pairwise comparison to explore significant main effects and interactions. Cohen’s *d* effect size was calculated to assess the magnitude of difference between each group. Values ranging from 0.20 to 0.49 indicated small, and values between 0.50 and 0.79 indicated medium. Values ranging from 0.80 to 1.29 indicated large, and values above 1.30 indicated very large effects. Values less than 0.20 were considered as having no noticeable effect [44]. The Pearson correlation coefficient was used to evaluate the degree of agreement between continuous variable. For all comparisons, significance was accepted at *p* < 0.050. All statistical analyses were performed using IBM SPSS Statistics 24 (IBM, Chicago, IL, USA).

## 3. Results

The analysis of demographic and clinical data were summarized in Table 1. Between Mid-age DM+ and Older DM+ groups, no difference was observed for gender, BMI, fall history, plantar numbness, prevalence of DPN, prevalence of PAD, or HbA1C values. Older people with diabetes had increased prevalence of high concern about falling and depression, but the difference didn’t reach statistical significance. The only clinical parameter reached statistical significance between Mid-age DM+ and Older DM+ was frailty prevalence (22% vs. 40%, *p =* 0.005). When comparing between Older DM− and Older DM+ groups, several clinical parameters reached statistical significance, including prevalence of high concern about falling, depression, and frailty. Furthermore, Table 1 illustrated that Mid-age HD+ group had higher prevalence of depression and frailty than Mid-age HD− group (29% vs. 27% and 24% vs. 20%, respectively). These prevalence were more prominent when comparing between Older HD+ and Older HD− (42% vs. 29% for depression and 58% vs. 23% for frailty).

Gait and balance performances for Older DM−, Mid-age DM+, and Older DM+ groups were summarized in Table 2. For comparison between older groups with and without diabetes, results were adjusted by age, gender, and BMI. All gait parameters reached statistical significance. In particular, Older DM+ group had significantly lower stride velocity and shorter stride length, as well as significantly longer gait cycle time and higher double support, when compared with Older DM− group (*d =* 1.06–1.73, *p* < 0.001). For balance performances, Older DM+ group had significantly larger ankle sway, hip sway, and center of mass sway than Older DM− group in double-stance test (*d =* 0.56–0.79, *p* < 0.010). When examining the aging impact on gait and balance among people with diabetes, results were adjusted by BMI. Compared to the Mid-age DM+ group, deteriorations were observed for all gait and balance parameters in Older DM+ group. Statistical significances were observed for the between-group difference of normalized stride length (*d =* 0.36, *p =* 0.029), gait cycle time (*d =* 0.34, *p =* 0.036), and double support (*d =* 0.46, *p =* 0.005), but not for stride velocity. In addition, aging induced deteriorations were more pronounced in challenging balance test (semi-tandem test, *d =* 0.31–0.45) than simple balance test (double-stance test, *d =* 0.27–0.31).

Gait and balance performances for Mid-age HD−, Mid-age HD+, Older HD−, and Older HD+ groups with adjustment by age, BMI, and maximum VPT value were summarized in Table 3. Among mid-age adults with diabetes, subjects undergoing HD had significantly deteriorated gait performances and lower body joints stability than HD− subjects (*d =* 0.63–1.73, *p* < 0.050). HD induced motor function deteriorations were more pronounced among older adults with diabetes, with larger effect size for each gait and balance parameter (*d =* 0.78–2.32, *p* < 0.050).

**Figure 3:**
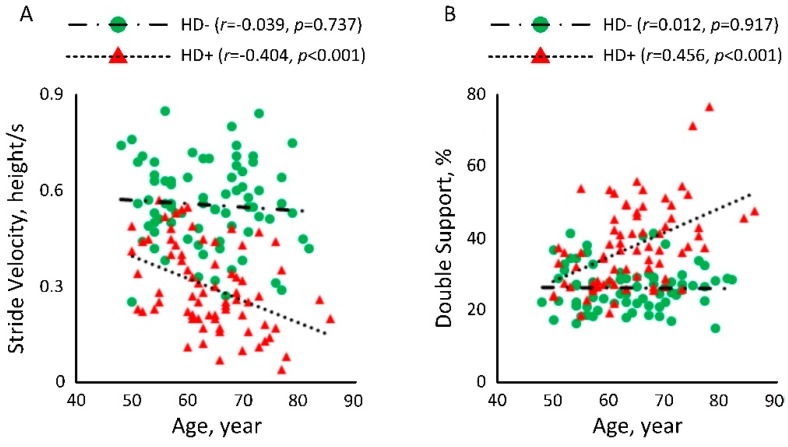
Correlation between age and (**A**) normalized stride velocity and (**B**) double support among people with diabetes with and without HD.

Figure 3 illustrated the correlation between age and gait performances among people with diabetes with and without HD. In Figure 3A, a significant negative correlation could be observed between age and normalized stride velocity among subjects undergoing HD (*r =* −0.404, *p* < 0.001). But the correlation among HD− subject was weak (*r =* −0.039, *p =* 0.737). Similarly, in Figure 3B, a significant correlation could be observed between age and double support among subjects undergoing HD (*r =* 0.456, *p* < 0.001). But the correlation among HD− subjects was weak (*r =* 0.012, *p =* 0.917).

**Figure 4:**
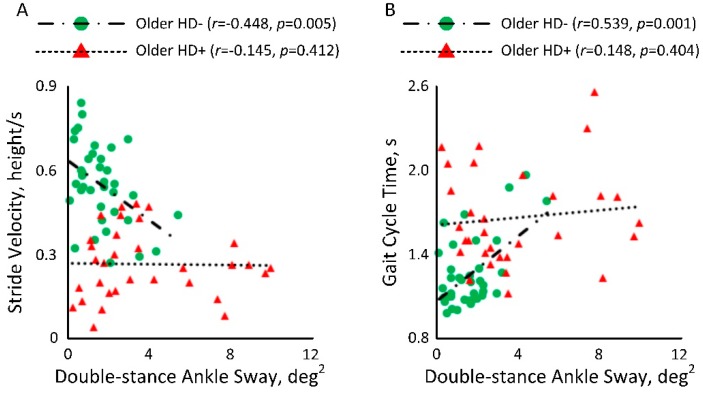
Correlation between double-stance ankle sway and (**A**) normalized stride velocity and (**B**) gait cycle time among older adults with diabetes with and without HD.

In Figure 4A, a significant negative correlation was observed between double-stance ankle sway and normalized stride velocity among older HD− (*r =* −0.448, *p =* 0.005). However, the correlation among older adults undergoing HD was weak (*r =* −0.145, *p =* 0.412). Figure 4B also showed a significant correlation between double-stance ankle sway and gait cycle time among older HD− (*r =* 0.539, *p =* 0.001). However, the correlation among older adults undergoing HD was weak (*r =* 0.148, *p =* 0.404).

## 4. Discussion

To our knowledge, this is the first study that objectively examined and quantified deteriorations in gait and balance among people with diabetes undergoing HD process, and compared with HD− people with diabetes as well as non-diabetic individuals. We were able to confirm our hypothesis that due to the impact of HD, this population have significantly worsened gait and lower body joints (ankle and hip) stability irrespective of age. In addition, motor function deterioration induced by HD is more pronounced among older adults than mid-age adults. A few previous studies have reported deteriorated gait and balance function of HD population when comparing with healthy controls [7,8,9], which is consistent with findings in this current study. However, none of previous studies compared HD population with cohorts with well-established model in motor function impairment, such as people with diabetes, as this current study did.

While gait and balance could be objectively quantified in a gait laboratory, such assessments are not practical for HD population. Many HD patients have limited mobility, suffer from post-dialysis fatigue, and rarely accept to go to different locations (including gait laboratory) than their regular HD clinics for the purpose of screening. Thus, prior studies were mostly limited to subjective or semi-objective tools (e.g., stopwatch-timed gait speed measurement) with limited information about gait performance [7]. While conventional assessment using force platform could be a suitable method to objectively quantify balance among HD population during routine clinic visit, force platform does not provide needed information to evaluate gait performance. In addition, force platform does not provide information about lower body joints stability (e.g., ankle or hip sway). To overcome these limitations, we used wearable sensors, which enabled us to quantify both gait and balance during regular dialysis clinic visit. The whole process of sensor attachment and administration of all gait and balance tests was less than 10 min with no reported fatigue, adverse event, or any other difficulty, making such measurements more practical and acceptable for routine screening among this vulnerable population. Such regular screening could capture early degradation caused by HD process and potentially assist with timely management/prevention of its consequences, including pre-mature frailty, falls, and foot problems (e.g., diabetic foot ulcers and joint deformities caused by alteration in biomechanics of lower extremity).

To our knowledge, this is the first study providing objective data to support motor function degradation among people with diabetes undergoing hemodialysis, which is noticeable from mid-age with further decline along with aging. Prior studies reported movement disorder among people with diabetes undergoing HD and linked it to diabetes-related conditions such as peripheral neuropathy and autonomy neuropathy, which are highly prevalent among HD population [45,46,47,48]. Our results suggested that alteration in gait performance (e.g., stride velocity, stride length, gait cycle time, and double support) among HD+ groups is not only related to diabetes, since higher degradation in gait was observed in HD+ compared to HD− (people with diabetes but no hemodialysis) among both mid-age and older adults. We speculate that gait deterioration among HD population is because of deconditioning related to HD process (approximate 4 h treatment for three times per week), which potentially leading to muscle weakness and joint rigidity. This speculation is supported by prior studies. For example, Jhamb et al. reported immobility and sedentary behavior caused by post-dialysis fatigue can accelerate motor function degradation [49]. The motor function deterioration among HD population beyond diabetes could be also explained by HD process itself. For instance, Floege et al. revealed that through the HD process, certain blood particulates are not able to easily pass through the filter and can accumulate in the body and form amyloid deposits in the joints, causing movement disorders [50]. Furthermore, our results revealed significant negative correlation between age and gait parameters of interest in HD+, suggesting that the negative impact of HD on gait is magnifying by aging. Along with aging, other age-related factors such as sensory alteration, sarcopenia, and cognitive decline could further deteriorate motor performance. 

Another interesting finding in this current study was that significant correlations were observed between lower body joints stability and gait among older HD−, while the correlations were weak in older individuals undergoing HD. Lattanzio et al. have shown that balance impairment was significantly associated with decline of kidney function, but gait impairment was not [51]. We speculate that diabetes and ESRD/HD conditions may cause different scales of impacts on gait and balance functions, leading to altered gait and balance performances. However this hypothesis needs to be validated in subsequent study.

Among metrics associated with balance control, our results suggested that ankle and hip sways are significantly higher among HD+ population compared to HD− population irrespective of age. No between-group difference was observed for CoM sway. Considering that higher prevalence of DPN among HD− groups in our sample (87% among HD− vs. 64% among HD+), we speculate that HD+ may benefit from reciprocal compensatory strategy (anti-phase motion of ankle and hip joints) to reduce the effect of ankle and hip sways on CoM. This is aligned with the report of Najafi et al. [42], which revealed that increase in CoM sway among DPN population is partially because of poorer reciprocal postural compensation compared to people without diabetes or neuropathy. Together we speculate that higher sways in ankle and hip joints among HD+ is mainly because of poorer muscle function caused by deconditioning among HD population and not necessarily because of diabetic neuropathy or poor sensory feedback. However, this needs to be confirmed in future studies.

In our study, we observed that Older HD+ group have a prevalence of frailty 53% higher than Older DM− group and 34% higher than Older HD− group. This demonstrated that ESRD and HD can magnify the likelihood of frailty, which can then lead to progression of adverse health outcomes, such as further motor function deterioration.

### Limitations

A major limitation of this study is that HD− groups were recruited from an outpatient podiatry clinic, and thus the majority had foot problems including DPN. The prevalence of DPN was higher among HD− groups than HD+ groups (87% among HD− vs. 64% among HD+). Therefore HD− groups may not represent general DM+ population. In addition, it is well established that DPN negatively affects gait and balance [52]. We believe, however, that this imbalance in DPN prevalence did not affect the conclusion of the study, since HD+ groups still had more deterioration than HD− groups irrespective of age. In addition, with adjusting by maximum VPT value (indicator of DPN severity), the between-group differences were still significant.

Our results also showed that HD− groups had higher prevalence of fall history and concern about falling, when compared to HD+ groups. This could be because of the high prevalence of DPN among HD− groups. Studies have shown that DPN has a high contribution to falls and fear of falling [53,54]. Another potential reason was that due to post-dialysis fatigue, subjects in HD+ groups were highly sedentary. Low level of daily physical activity in individuals undergoing hemodialysis [55] may lead to low prevalence of fall history and concern about falling.

Finally, we noticed that HD+ groups had significantly lower Hb1AC level than HD− groups. In our previous study, we demonstrated that higher Hb1AC level is correlated with poorer balance [40]. Thus, we anticipate that lower HbA1C observed in HD+ groups will not affect the significance of between-group difference observed in this study. On the other hand, it is debated whether HbA1C is a reliable metric to determine glucose level among HD patients [56]. In other words, Hb1Ac level is calculated by measuring hemoglobin to which glucose is bound in red blood cells (RBCs). While the longer an individual’s RBCs are in circulation the greater chance they will be glycosylated. The average lifespan of RBCs is about 120 days in healthy individuals [57]. However, the RBCs lifespan in patients with ESRD can reduce by 30% to 70% [58]. Therefore, the Hb1Ac level could be systematically lower in patients with ESRD. In addition, study has shown that sevelamer carbonate, which is often used in individuals undergoing hemodialysis to control their phosphorus levels [59], can significantly reduce HbA1c level [60]. Because of these limitations of Hb1A1C measurement among HD patients, we didn’t adjust the results by Hb1A1C level.

## 5. Conclusions

In conclusion, while diabetes deteriorates gait and lower body joints stability, HD magnifies the deterioration beyond diabetes condition irrespective of age. In addition, progression in age significantly affects the magnitude of gait and lower body joints stability deterioration among HD patients, when compared with HD− individuals. Results revealed that poor lower body joints stability is correlated with poor gait in Older HD− group. However, interestingly, no correlation was observed between poor lower body joints stability and poor gait among HD+ group and the deterioration of gait is highly depends on age. This study demonstrated the feasibility of using wearable sensors to quantify gait and balance as a part routine patient visit for HD population. Such assessment may assist early detection of motor function decline and thus promote early intervention.

## Figures and Tables

**Table 1:** General characteristics of the study groups.

Variable	Older Adults without Diabetes (DM−)	People with Diabetes (DM+)						*p*-Value	
		Mid-Age Adults			Older Adults			Older DM− vs. Older DM+	Mid-age DM+ vs. Older DM+ *
		Total	HD−	HD+	Total	HD−	HD+		
**Subject Number, n**	45	78	40	38	73	37	36	-	-
**Age, years (mean ± SD)**	73.4 ± 6.8	57.2 ± 4.2	56.5 ± 4.2	58.1 ± 4.1	71.4 ± 5.4	71.3 ± 4.6	71.5 ± 6.1	0.073	**<0.001**
**Female, %**	71%	51%	55%	47%	52%	49%	56%	**0.041**	0.924
**BMI, kg/m^2^ (mean ± SD)**	27.1 ± 5.0	31.1 ± 7.1	31.2 ± 6.1	31.1 ± 8.2	30.8 ± 5.9	29.9 ± 5.2	31.8 ± 6.5	**<0.001**	0.780
**Fall History, %**	29%	36%	51%	21%	28%	36%	22%	0.951	0.307
**High Concern about Falling, %**	36%	65%	80%	50%	74%	78%	69%	**<0.001**	0.230
**Depression, %**	13%	28%	27%	29%	36%	29%	42%	**0.005**	0.246
**Frailty, %**	5%	22%	20%	24%	40%	23%	58%	**<0.001**	**0.005**
**Plantar Numbness, VPT (mean ± SD)**	-	32.0 ± 9.8	34.6 ± 8.9	29.4 ± 10.2	32.0 ± 10.1	35.0 ± 8.5	29.1 ± 10.7	-	0.982
**Diabetic Peripheral Neuropathy, %**	-	76%	85%	66%	74%	88%	60%	-	0.845
**Peripheral Artery Disease, %**	-	59%	57%	61%	63%	64%	63%	-	0.650
**HbA1c, % (mean ± SD)**	-	7.2 ± 2.2	7.9 ± 2.8	6.6 ± 1.5	7.0 ± 1.6	7.2 ± 2.0	6.8 ± 1.2	-	0.574

BMI: Body-mass-index. VPT: Vibration Perception Threshold. *: *p*-value calculated for Total Older DM+ and Total Mid-age DM+. Significant difference between groups were indicated in bold.

**Table 2:** Between-group comparison for gait and balance performance among Older DM−, Mid-age DM+, and Older DM+ groups.

		Older DM− n = 45	Mid-Age DM+ n = 78	Older DM+ n = 73	Mid-Age DM+ vs. Older DM−			Older DM+ vs. Older DM−			Older DM+ vs. Mid-Age DM+		
					Diff (%)	*p*-Value *	*d* *	Diff (%)	*p*-Value ^†^	*d* ^†^	Diff (%)	*p*-Value ^‡^	*d* ^‡^
**Gait**	**Stride Velocity, m/s (mean ± SD)**	1.14 ± 0.17	0.75 ± 0.29	0.68 ± 0.36	−34%	**<0.001**	1.55	−40%	**<0.001**	1.61	−10%	0.171	0.22
	**Normalized Stride Velocity, height/s (mean ± SD)**	0.70 ± 0.10	0.45 ± 0.17	0.40 ± 0.20	−36%	**<0.001**	1.62	−43%	**<0.001**	1.73	−11%	0.091	0.27
	**Stride Length, m (mean ± SD)**	1.23 ± 0.14	0.98 ± 0.31	0.89 ± 0.34	−20%	**<0.001**	1.02	−28%	**<0.001**	1.36	−10%	0.071	0.29
	**Normalized Stride Length, % height (mean ± SD)**	75.43 ± 7.14	59.42 ± 17.08	53.04 ± 18.42	−21%	**<0.001**	1.11	−30%	**<0.001**	1.53	−11%	**0.029**	0.36
	**Gait Cycle Time, s (mean ± SD)**	1.10 ± 0.11	1.39 ± 0.24	1.53 ± 0.52	26%	**<0.001**	1.34	38%	**<0.001**	1.06	10%	**0.036**	0.34
	**Double Support, % (mean ± SD)**	22.66 ± 4.76	29.85 ± 8.94	34.92 ± 13.62	32%	**<0.001**	0.93	54%	**<0.001**	1.09	17%	**0.005**	0.46
**Balance Double-Stance**	**Ankle Sway, deg^2^ (mean ± SD)**	0.81 ± 0.75	2.24 ± 2.12	2.95 ± 3.09	177%	**<0.001**	0.86	264%	**<0.001**	0.79	32%	0.105	0.27
	**Hip Sway, deg^2^ (mean ± SD)**	0.94 ± 0.80	2.15 ± 2.43	3.15 ± 4.54	129%	**0.005**	0.57	235%	**0.008**	0.56	46%	0.098	0.27
	**CoM Sway, cm^2^ (mean ± SD)**	0.16 ± 0.11	0.27 ± 0.24	0.36 ± 0.36	69%	**0.023**	0.47	125%	**0.002**	0.68	33%	0.076	0.30
	**Normalized CoM Sway, cm^2^/height (mean ± SD SD)**	0.10 ± 0.07	0.16 ± 0.14	0.22 ± 0.22	60%	**0.022**	0.47	120%	**0.002**	0.67	38%	0.065	0.31
**Balance Semi-Tandem**	**Ankle Sway, deg^2^ (mean ± SD)**	-	2.44 ± 2.34	3.67 ± 4.14	-	-	-	-	-	-	51%	**0.044**	0.38
	**Hip Sway, deg^2^ (mean ± SD)**	-	2.32 ± 2.40	3.50 ± 3.73	-	-	-	-	-	-	51%	**0.034**	0.40
	**CoM Sway, cm^2^ (mean ± SD)**	-	0.29 ± 0.29	0.74 ± 1.48	-	-	-	-	-	-	150%	**0.017**	0.45
	**Normalized CoM Sway, cm^2^/height (mean ± SD)**	-	0.18 ± 0.17	0.23 ± 0.21	-	-	-	-	-	-	28%	0.100	0.31

CoM: Center of Mass. *: Results were adjusted by gender and BMI. ^†^: Results were adjusted by age, gender, and BMI. ^‡^: Results were adjusted by BMI. Significant difference between groups were indicated in bold. Effect sizes were calculated as Cohen’s *d*.

**Table 3:** Between-group comparison for gait and balance performance among Mid-age HD−, Mid-age HD+, Older HD−, and Older HD+ groups.

		Mid-Age DM+					Older DM+				
		HD− n = 40	HD+ n = 38	Diff (%)	*p*-Value *	*d* *	HD− n = 37	HD+ n = 36	Diff (%)	*p*-Value *	*d* *
**Gait**	**Stride Velocity, m/s (mean ± SD)**	0.93 ± 0.22	0.55 ± 0.22	−41%	**<0.001**	1.68	0.96 ± 0.27	0.40 ± 0.20	−58%	**<0.001**	2.31
	**Normalized Stride Velocity, height/s (mean ± SD)**	0.56 ± 0.13	0.34 ± 0.13	−39%	**<0.001**	1.68	0.55 ± 0.15	0.25 ± 0.12	−55%	**<0.001**	2.14
	**Stride Length, m (mean ± SD)**	1.17 ± 0.20	0.78 ± 0.26	−33%	**<0.001**	1.67	1.15 ± 0.22	0.62 ± 0.23	−46%	**<0.001**	2.32
	**Normalized Stride Length, % height (mean ± SD)**	69.96 ± 10.01	48.11 ± 15.71	−31%	**<0.001**	1.73	66.04 ± 11.47	39.50 ± 14.29	−42%	**<0.001**	2.11
	**Gait Cycle Time, s (mean ± SD)**	1.29 ± 0.20	1.49 ± 0.24	15%	**0.001**	0.83	1.26 ± 0.24	1.80 ± 0.60	43%	**<0.001**	1.15
	**Double Support, % (mean ± SD)**	26.30 ± 6.37	33.75 ± 9.67	28%	**<0.001**	0.87	26.34 ± 5.50	43.74 ± 14.22	66%	**<0.001**	1.42
**Balance Double-Stance**	**Ankle Sway, deg^2^ (mean ± SD)**	1.48 ± 0.90	3.03 ± 2.68	105%	**0.002**	0.76	1.54 ± 1.04	4.40 ± 3.82	187%	**<0.001**	1.19
	**Hip Sway, deg^2^ (mean ± SD)**	1.29 ± 1.02	3.03 ± 3.09	134%	**0.003**	0.72	1.55 ± 1.35	4.91 ± 5.97	217%	**0.001**	0.90
	**CoM Sway, cm^2^ (mean ± SD)**	0.27 ± 0.21	0.28 ± 0.28	5%	0.743	0.08	0.35 ± 0.26	0.37 ± 0.44	6%	0.248	0.30
	**Normalized CoM Sway, cm^2^/height (mean ± SD)**	0.16 ± 0.12	0.17 ± 0.17	6%	0.708	0.09	0.20 ± 0.15	0.23 ± 0.27	15%	0.159	0.37
**Balance Semi-Tandem**	**Ankle Sway, deg^2^ (mean ± SD)**	1.66 ± 1.13	2.99 ± 2.78	80%	**0.025**	0.63	1.73 ± 1.78	4.85 ± 4.79	180%	**0.016**	0.78
	**Hip Sway, deg^2^ (mean ± SD)**	1.21 ± 0.71	3.10 ± 2.81	157%	**0.014**	0.70	1.65 ± 1.63	4.63 ± 4.27	181%	**0.002**	1.01
	**CoM Sway, cm^2^ (mean ± SD)**	0.31 ± 0.19	0.29 ± 0.35	−6%	0.709	0.11	0.37 ± 0.33	0.37 ± 0.37	0	0.483	0.23
	**Normalized CoM Sway, cm^2^/height (mean ± SD)**	0.18 ± 0.21	0.18 ± 0.35	<1%	0.639	0.13	0.21 ± 0.19	0.24 ± 0.23	14%	0.339	0.31

CoM: Center of Mass. *: Results were adjusted by age, BMI, maximum VPT. Significant difference between groups were indicated in bold. Effect sizes were calculated as Cohen’s *d*.
